# A Primer on the Role of Boredom in Self-Controlled Sports and Exercise Behavior

**DOI:** 10.3389/fpsyg.2021.637839

**Published:** 2021-03-01

**Authors:** Wanja Wolff, Maik Bieleke, Corinna S. Martarelli, James Danckert

**Affiliations:** ^1^Department of Sport Science, University of Konstanz, Konstanz, Germany; ^2^Department of Educational Psychology, University of Bern, Bern, Switzerland; ^3^Department for Psychology of Development and Education, Faculty of Psychology, University of Vienna, Vienna, Austria; ^4^Faculty of Psychology, Swiss Distance University Institute, Brig, Switzerland; ^5^Department of Psychology, University of Waterloo, Waterloo, ON, Canada

**Keywords:** boredom, self-control, effort, reward-based choice, exercise, sports, decision making

## Abstract

Self-control is critical for successful participation and performance in sports and therefore has attracted considerable research interest. Yet, knowledge about self-control remains surprisingly incomplete and inconsistent. Here, we draw attention to boredom as an experience that likely plays an important role in sports and exercise (e.g., exercise can be perceived as boring but can also be used to alleviate boredom). Specifically, we argue that studying boredom in the context of sports and exercise will also advance our understanding of self-control as a reward-based choice. We demonstrate this by discussing evidence for links between self-control and boredom and by highlighting the role boredom plays for guiding goal-directed behavior. As such, boredom is likely to interact with self-control in affecting sports performance and exercise participation. We close by highlighting several promising routes for integrating self-control and boredom research in the context of sports performance and exercise behavior.

## Introduction

Although self-control is a heavily researched psychological concept, an inconsistent body of literature limits the understanding of self-control’s role in orienting goal-directed behavior (e.g., sports and exercise). Recently, it has been argued that boredom might be an overlooked confound in self-control research that has contributed to some of the inconsistencies in this research area. Since exercise can be perceived as boring, but can also be used to alleviate boredom, boredom is likely to play an important role in the context of sports and exercise. However, sports psychological research has rarely turned to boredom as an important factor to examine. This perspective aims to address this gap and to explicate how boredom and self-control are expected to interact in orienting goal-directed behavior. As self-control has received substantial research interest already, we will only briefly explicate the current understanding of self-control as a reward-based choice and devote more space to boredom. Specifically, we will highlight why boredom might have acted as an overlooked confound in self-control research, and how it might have affected self-controlled sports and exercise behavior (directly and via its interaction with self-control). We close by highlighting several promising routes for integrating self-control and boredom research in the context of sports performance and exercise behavior.

### Self-Control in Sports and Exercise

The man who can drive himself further once the effort gets painful is the man who will win.

– Sir Roger Bannister (the first human to run a mile in under 4 min)

The importance of self-control in the context of sports and exercise is widely acknowledged (Englert and Taylor, 2021). A plethora of studies have highlighted the relevance of self-control for regular exercise (Hagger et al., 2010b; de Ridder et al., 2012) and for performing well in a sporting task (Giboin and Wolff, 2019; Brown et al., 2020). The importance of self-control—and its resulting popularity as a topic for research in sports and exercise psychology—is intuitively appealing. After all, self-control is defined as “the efforts people exert to stimulate desirable responses and inhibit undesirable responses” (de Ridder et al., 2012, p. 77) and the notion of physical and/or mental effort is inherent to sports and exercise. In addition, self-control is often also referred to as *willpower* (Ainslie, 2020), a quality that is held in exceptionally high regard in sports (as exemplified in the above quote from Sir Roger Bannister).

It is important to note that applying self-control seems to carry an intrinsic cost (Kool and Botvinick, 2013; Ackerman et al., 2020) whose origin is still subject to debate (Shenhav et al., 2017). Attesting to this costliness, the application of self-control is tightly coupled with the sensation of effort that people normally try to avoid (Shenhav et al., 2017). Accordingly, a large body of research indicates that the application of self-control in one task will lead to impaired performance in a subsequent self-control demanding task (Hagger et al., 2010a; Giboin and Wolff, 2019; Brown et al., 2020). For example, after having applied self-control to complete a challenging work assignment, one might struggle to muster the self-control needed to go out for a late run in the dark. Current theorizing conceptualizes self-control application as a reward-based choice (Kurzban et al., 2013; Shenhav et al., 2013, 2017; Wolff and Martarelli, 2020) and from this perspective, the self-control costs of going out for a late run can outweigh the run’s prospective benefits for the aspiring exerciser. Crucially, this theorizing implies that not mustering the self-control that would be required to engage (or continue) with a self-control demanding activity may not necessarily reflect a failure of self-control but might simply reflect an adaptive reward-based choice to switch from exploitation to exploration behavior (Bieleke and Wolff, in press). In line with this idea, empirical and theoretical work indicates that incurred self-control costs make people less willing to apply further self-control (Wolff and Martarelli, 2020), particularly if goal progress is not obvious to them (Osgood, 2018).

In experimental sports psychology research, the effect of prior self-control exertion on subsequent sports performance is typically investigated with a sequential two-task paradigm (Englert, 2016). Here, an experimental group performs a high self-control task (HCT), and a control group performs a low self-control task (LCT) after which both groups perform a self-control demanding sporting task (e.g., dart throwing, sprint starts, or isometric strength endurance). An example of a frequently used HCT in self-control research is the incongruent Stroop task (Wolff et al., 2018). Attesting to its self-control demands, the incongruent Stroop is generally associated with higher error rates and longer response latencies than its congruent counterpart (which is frequently used as the LCT in self-control research) and is perceived as more self-control demanding (Wolff et al., 2019). If the prior application of self-control indeed reduces the willingness to invest further effort (Wolff and Martarelli, 2020), then participants should perform worse in the sporting task after an HCT than after an LCT.

Indeed, recent meta-analytic evidence provides support for this hypothesis (Giboin and Wolff, 2019; Brown et al., 2020). However, the magnitude of performance impairment is not robustly linked to the duration of the prior self-control task (Giboin and Wolff, 2019), which conflicts with the theoretical proposition that the magnitude of the performance decrement should scale linearly with the duration of the prior self-control task (Hagger et al., 2010a). In addition, a recent bias-sensitive meta-analysis of the literature suggests that initial estimates of the effects of prior self-control on subsequent sports performance might be smaller than initially assumed (Holgado et al., 2020). This inconsistent body of literature limits our understanding of the relationship between self-control and sports performance.

### Boredom: A Possible Confound

Outside the sporting context, self-control research with the sequential two-task paradigm has yielded similarly heterogeneous findings (Wolff et al., 2018). In fact, null findings (Wolff et al., 2019), evidence for publication bias (Carter and McCullough, 2014), and a large file-drawer of unpublished studies (Wolff et al., 2018) in this field have prompted researchers to conclude that it is still unclear if prior self-control exertion robustly impairs subsequent self-control performance (Friese et al., 2017).

Recently, it has been suggested that boredom might have contributed to these inconsistencies by acting as a confound of self-control effects on performance (Wolff and Martarelli, 2020). Indeed, it is plausible that LCTs and HCTs not only differ with respect to the self-control demands they impose but also in regard to how boring they are perceived to be (Milyavskaya et al., 2019). It seems particularly likely that some LCTs are systematically more boring than their HCT counterparts because they are designed to place minimal demands on self-control (and indeed, cognitive processes), thereby rendering them prototypical boredom inductions (Wolff and Martarelli, 2020). Accordingly, one recent study has shown that a more self-control demanding modified version of the Stroop task was perceived as less boring than a traditional Stroop (Bieleke et al., 2020a). Further attesting to the importance of boredom in self-control research, another study showed that if an HCT created feelings of boredom, performance on a subsequent self-control task was impaired (Osgood, 2015). Conversely, a very recent study has shown that if a primary LCT was perceived as boring, no performance differences between the LCT and HCT conditions could be observed in a subsequent isometric endurance task (Mangin et al., under review). The potential for boredom in LCT is made even more plausible by the fact that tasks that are typically used in self-control research are also frequently used in boredom research as experimental inductions of boredom (Wolff and Martarelli, 2020).

Boredom might also play a role in explaining the absence of a robust correlation between prior self-control task duration and any subsequent drop in performance. Neither boredom nor task-imposed self-control demands should be treated as static experiences (Mills and Christoff, 2018; Wolff and Martarelli, 2020). A task that was initially very demanding in terms of self-control might become progressively easier to perform due to learning or practice effects. To illustrate, while a tennis serve might be a very complex task for a beginner, professional tennis players have practiced them ad nauseam, making the movement execution second nature to them. Thus, an HCT can turn into an LCT as a function of practice. In line with this, one recent high-powered study showed that performance improved over time on an incongruent Stroop task, the longer participants worked on it (Wolff et al., 2019). Analogous arguments can be made with regard to boredom, which is best understood as a highly dynamic feeling state (Mills and Christoff, 2018) that varies greatly as a function of the task characteristics (e.g., different task demands; Bieleke et al., 2020a). Thus, neglecting the temporal dynamics of boredom and self-control demands might contribute to the inconsistent link between prior task duration and subsequent performance decrement (Wolff and Martarelli, 2020).

### Exercise Can Be Boring

Marathon running is a terrible experience: monotonous, heavy, and exhausting.

– Veikko Karvonen (Olympic medalist at the 1956 marathon)

Crucially, boredom is not only a potential confound in research on self-control demanding laboratory tasks. Besides being inherently linked to effort, engaging in sports and exercise can also be plain boring (as exemplified by the quote from Veiko Karvonen above). For instance, boredom has recently been identified as a frequent obstacle during aerobic endurance exercises (Hirsch et al., 2020). On the other hand, sports and exercise can also be used to alleviate boredom (Morris et al., 2003). Thus, boredom clearly plays a multifaceted role in the sports and exercise context. Indeed, the relevance of boredom in sports had already been acknowledged as early as 1926, when the monotony of regular athletic training was introduced as an analogy of industrialized work (Davies, 1926). However, despite an abundance of lay intuition on the detrimental effects boredom has on sports performance and exercise behavior (Orenstein, 2012), research that has specifically assessed boredom in sports and exercise remains scarce. One recent notable exception showed that even professional athletes frequently struggle with boredom, with detrimental consequences for performance (Velasco and Jorda, 2020). In the exercise domain, boredom proneness has been associated with less self-reported vigorous exercise behavior (Wolff et al., 2020a).

One very likely reason for this research gap is that research from sports and exercise has primarily focused on the self-control demands of completing effortful and difficult tasks, like performing a sprint start (Englert et al., 2015), persisting for as long as possible in an endurance task (Boat et al., 2020), or adhering to an exercise regimen (Englert and Rummel, 2016). This focus makes intuitive sense, since self-control is per definition linked to the notion of effort (de Ridder et al., 2012). However, this also neglects self-control demands that do not fit the prototypical mold of high demand and high effort tasks (e.g., practicing basketball free throw technique ad infinitum). In the second part of this perspective, we will explicate that boredom is one such demand and highlight how it is intrinsically related to self-control and how it uniquely affects behavior.

## Arguments for Boredom as an Important Factor in Self-Controlled Sports and Exercise Behavior

Another reason for the scarcity of boredom research in sports is that until very recently boredom had not attracted much scientific interest in general (Mills and Christoff, 2018). This is rapidly changing, however, Recent work has advanced boredom research by providing more definitional clarity for both the state (Bench and Lench, 2013; Elpidorou, 2018; Elpidorou, 2020) and what it means to be boredom prone as a trait (Tam et al., under review). This work has clarified the conditions under which state boredom is likely to occur (Westgate and Wilson, 2018), as well as its functional relevance (Bench and Lench, 2019; Wolff and Martarelli, 2020). Finally, recent work has linked trait boredom proneness and self-control and explicated their joint role in goal-directed behavior (Wolff and Martarelli, 2020). These recent conceptual and empirical advancements provide an excellent starting point for investigating the role of boredom—at the level of both state and trait—in the context of self-controlled sports and exercise behavior.

### Boredom and Self-Control Overlap by Definition

Boredom has been defined as the “aversive state that occurs when we are not able to successfully engage attention” and an “awareness of a high degree of mental effort expended in an attempt to engage with the task (Eastwood et al., 2012, p. 481)”. Accordingly, boredom differs from seemingly related states (like low interest or amotivation), by being a decidedly aversive sensation where one wants to engage with something but is unable to do so (Mugon et al., 2018; Danckert and Eastwood, 2020). In line with the latter, recent research has shown that performing an easy but boring task creates sensations of fatigue that can even outweigh the fatigue that is experienced by performing a demanding self-control task (Milyavskaya et al., 2019). Thus, inherent to their respective definitions, boredom and self-control share two core features. The capacity to control attention has been identified as the most important function of self-control (Schmeichel and Baumeister, 2010) and failure to engage attention with available activities accompanies the sensation of boredom (Malkovsky et al., 2012; Hunter and Eastwood, 2018). With respect to effort, applying self-control creates the sensation of effort. Likewise, any task that leads to the feeling of boredom requires mental effort if we decide to redouble our efforts to stick with the boring task. Consequently, it has been proposed that boredom affects results of self-control research because staying engaged with a boring task constitutes a self-control demand (Milyavskaya et al., 2019; Wolff and Martarelli, 2020). Thus, although an LCT is expected to be less self-control demanding than an HCT in terms of its task-specific self-control demands, this effect might be offset if the LCT leads to more boredom than the HCT, which would unintentionally increase the self-control demands of continuing to work on the LCT. In the same vein, a slow ten-kilometer run might be self-control demanding not (only) because the runner has to regulate aversive exercise-induced bodily sensations but also because the run itself has become boring to the runner.

### Boredom in Sports and Exercise

Another perspective on why boredom likely matters for sports and exercise comes from research on the conditions under which boredom occurs. Research shows that boredom can result from an incompatibility between the demands associated with a current activity and the available attentional resources (Pekrun et al., 2010; Eastwood et al., 2012; Westgate and Wilson, 2018). Critically, this attentional mismatch can occur when an activity is underchallenging (e.g., running at moderate intensities) or overchallenging (e.g., trying to dunk a basketball although one can clearly not jump high enough) (Pekrun et al., 2010). In addition, boredom can occur when an activity is perceived as being void of meaning (van Tilburg and Igou, 2012, van Tilburg and Igou, 2017; van Tilburg et al., 2013). It makes intuitive sense that perceived lack of meaning might be important in the context of exercise behavior. To illustrate, exercisers often do strength-related exercises at the gym or go running in the forest not because they genuinely like it but because they hope this will improve appearance or reduce weight (DiBartolo et al., 2007). However, the gains made by dragging oneself to the gym or the forest accumulate only very slowly, thereby making it easy to lose faith in the meaningfulness of this behavior. Beyond a lack of perceived meaning, athletes might perceive some exercises as boring because they do not yield immediate feedback and rewards. To illustrate, athletes who genuinely like the sport they engage in might not enjoy the ancillary training they have to carry out in order to do well at their main sports. In line with this, unpublished pilot data from our lab provide strong statistical evidence that team sports athletes perceive their ancillary individual training sessions to be more boring than their primary sports-specific training sessions (for further information, please see OSF | Perspective on Boredom and Self-Control in Sports). One possible reason for this could be that ancillary individual training sessions are perceived as less rewarding (e.g., in terms of enjoyment) and are less rich in relevant feedback (e.g., relevance of subjectively improved running efficiency for getting better at scoring goals in soccer) than the training sessions of their primary sports. Taken together, the conditions that are conducive to boredom are likely to occur frequently in various sports and exercise settings.

Critically, while sports and exercise are associated with heightened arousal, boredom has traditionally been associated with low arousal (Mikulas and Vodanovich, 1993). At first glance, this is at odds with the premise of this paper. However, as the examples above indicate, boredom is also likely to occur when arousal is high (e.g., when doing endurance training as a soccer player). In line with this, research shows that boredom can be considered a mixed arousal state (Merrifield and Danckert, 2014) or even a high arousal state (Danckert et al., 2018). One potential response to this mix of results regarding the physiological signature of boredom is to suggest that arousal should not be considered a key feature of the definition of boredom (Elpidorou, 2020). While this debate cannot be resolved here, it does suggest that while arousal is an important factor to consider in the proposed research program, it should not be considered integral to it.

### Boredom Is a Signal to Change Behavior

Importantly, although boredom is an aversive sensation, it is still assumed to have high functional relevance (analogous to the experience of pain; Danckert and Eastwood, 2020). It has been proposed that boredom’s function is to signal that one is failing to engage with an ongoing activity and/or that one could be deploying one’s attention toward more rewarding activities. Thus, by tracking diminishing returns of an ongoing activity (Berlyne, 1970) and by increasing sensitivity to more rewarding alternatives (Milyavskaya et al., 2019) boredom acts as a catalyst for behavioral change (Bieleke and Wolff, in press; Martarelli and Wolff, 2020; Wolff and Martarelli, 2020). This understanding of boredom’s function has been supported by recent computational (Gomez-Ramirez and Costa, 2017) and empirical work (Geana et al., 2016). This has the important implication that despite its negative connotation, the state of boredom is neither good nor bad *per se*, its aversiveness merely acts as a signal to do *something* else. What this *something* will be seems to depend on situational constraints. More specifically, recent work has shown that getting bored during a hedonically negative experience instigates a switch to hedonically positive experiences, and, interestingly, the opposite is also true: boredom during hedonically positive experiences appears to instigate a switch to hedonically negative experiences (Bench and Lench, 2019). Thus, boredom urges one to seek out an experience that is different to one’s current experience. For example, a runner might get bored because of the monotony of the exercise and feels the urge to do something more exciting. Likewise, getting bored by a tiresome work assignment could also drive one to go to the gym and engage in a more energizing activity.

### Self-Control and Boredom Are Tightly Associated

**FIGURE 1:**
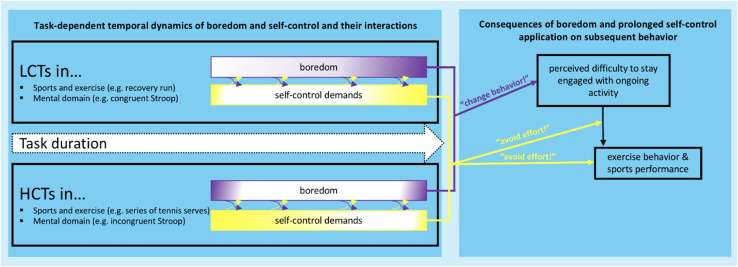
Working model on the proposed interplay of boredom and self-control in modulating exercise behavior and sports performance (model adapted from Martarelli and Wolff, 2020; Wolff and Martarelli, 2020). The left panel visualizes the temporal dynamics of task-induced boredom and self-control demands as a function of the type of task [low control task (LCT); vs. high control task (HCT)] and the duration of the task. More specifically, the model proposes that task characteristics change as a function of task duration which in turn leads to changes in task-imposed self-control demands and task-induced boredom. The color gradients reflect the potential changes in respective signal strength as a function of time on task. For example, an LCT (e.g., recovery run) that becomes monotonous and under-stimulating over time, might become more boring over time, *and* to keep going despite being bored increases the run’s self-control demands. On the other hand, HCTs can lead to the experience of boredom at the beginning due to over-stimulation (e.g., tennis serves executed by a novice) and at the end due to under-stimulation (e.g., as a result of overlearning). The left panel is a schematic representation of the assumed temporal dynamics and the arrows that connect boredom and self-control demands indicate that both signals are expected to affect each other. Importantly, the temporal dynamics of each sensation must not be linear and are assumed to depend on task characteristics and individual differences (for a comprehensive version of the model and an in-depth discussion, please see Wolff and Martarelli, 2020). The right panel visualizes the behavioral relevance of boredom and self-control exertion in affecting sports performance and exercise behavior. Boredom signals whether one should explore more rewarding alternative activities (“change behavior!”) and self-control demands signal whether one should avoid investing further effort (“avoid effort!”). Thus, at any given time during the task (visualized by the arrow on the left panel), those signals are expected to vary in strength. The model proposes that boredom has a direct effect on the perceived difficulty to stay engaged with an ongoing activity, which in turn impacts ongoing exercise behavior and sports performance. Successful participation and performance in sports relies on self-control; therefore, the model proposes that self-control moderates the effect of perceived difficulty on exercise behavior and sports performance. Crucially, other variables are likely to influence the proposed relationships, such as differences in perceived meaning and trait boredom or trait self-control might moderate the velocity of gradient changes over time.

Boredom and the sense of effort that accompanies the application of self-control seem to affect goal-directed behavior in close tandem, but with clearly differentiable functions (Figure 1). As we have outlined above, applying self-control creates the sensation of effort and empirical evidence indicates that this reduces the willingness to invest further effort (Sjåstad and Baumeister, 2018; Lin et al., 2020). Recent theorizing postulates that self-control allocation reflects the reward-based choice that the expected value of applying control outweighs the resulting self-control costs (Shenhav et al., 2013, 2017). In this conceptualization, the sensation of effort assumes the role of tracking the ongoing control costs and biasing behavior away from further application of self-control processes (Kurzban et al., 2013; Shenhav et al., 2013; Wolff and Martarelli, 2020).

Similarly, boredom’s function is to instigate a change in behavior that is driven by a reduced valuation of current task value (Berlyne, 1970) and a greater sensitivity for rewards (Milyavskaya et al., 2019). Thus, boredom uniquely affects goal pursuit by instigating a change in behavior. Crucially, boredom can also make goal pursuit more self-control demanding. As a task becomes boring, signaling the urge to do something else, we must choose whether to engage a different task or increase our efforts to persist with the current one. Choosing the latter course of action (which is likely prevalent in the context of sports and exercise where athletes choose to persist on monotonous training regimes), directly contributes to rising self-control costs that, according to recent reward-based models of self-control (Kurzban et al., 2013; Shenhav et al., 2013, 2017; Wolff and Martarelli, 2020), people would normally strive to minimize. Thus, from a conceptual point of view, boredom and self-control appear to be tightly coupled in their guiding function for goal-directed behavior (Wolff and Martarelli, 2020; Bieleke and Wolff, in press).

In line with this, empirical evidence points toward a strong inverse relationship between trait self-control and boredom proneness (Mugon et al., 2018). This implies that individuals who experience the state of being bored frequently and intensely (Tam et al., under review) (e.g., in the face of repetitive gym work) should also exhibit lower levels of self-control, making it difficult for them to cope with the boredom-induced urges to disengage and instead apply the required self-control to persist with the boring task. Attesting to the existence of such a link in the exercise context, one recent study showed that high trait self-control and low boredom proneness form part of a latent personality profile that was linked to more regular exercise, whereas a profile with lower self-control and higher boredom proneness was linked with considerably lower exercise levels (Wolff et al., 2020a). Further evidence on the proposed interplay between boredom proneness and self-control comes from recent research on adherence to the social distancing guidelines amidst the ongoing COVID-19 pandemic (Bieleke et al., 2020b; Boylan et al., 2020; Wolff et al., 2020b; Martarelli et al., 2021). In line with the above propositions, boredom proneness was linked to less adherence and this effect was mediated by the perceived difficulty to comply with social distancing guidelines (Wolff et al., 2020b). In addition, high self-control was linked to better adherence and moderated the link between the perceived difficulties and adherence behavior.

The close relationship between boredom and self-control is further exemplified by both concepts’ link to changes in the perception of the passage of time (Vohs and Schmeichel, 2003; Danckert and Allman, 2005). For example, while boredom proneness has been linked with the tendency to perceive time as running slowly, the opposite was found for high trait self-control (Witowska et al., 2020). On the state level, experiencing boredom and applying self-control (along with the sensation of effort this creates) has been linked to overestimating the time spent on a boring and/or self-control demanding task (Vohs and Schmeichel, 2003; Danckert and Allman, 2005). Importantly, biases in time perception might directly affect the cost-benefit analysis that underlies goal-directed behavior (Kurzban et al., 2013; Ainslie, 2020).

## Discussion

Up to now, we have made the case for the overlooked importance of boredom in the context of sports and exercise. In the conclusion of this paper, we outline some key implications and avenues for integrating boredom into research on self-control in sports and exercise behavior more generally.

Experimental research on the role of prior mental exertion on subsequent sports performance, as well as research on the role of self-control for exercise adherence should systematically assess state and trait boredom. As outlined above, the experience of boredom might alter sports performance in its own right (e.g., making it feel aversive) and could alter task-induced self-control demands (e.g., making it feel more demanding). Considering boredom would potentially help to understand and dissolve inconsistent research findings. With respect to the latter, boredom prone individuals might find (certain) exercises harder to adhere to. This seems more likely for some kinds of sport or activities than for others (e.g., repetitions in the gym may be more prone to ratings of boredom, whereas adventure sports may be chosen as an *escape* from boredom; Kerr and Houge Mackenzie, 2012). Likewise, it is plausible that the specific settings in which a sport or exercise is embedded in (e.g., collective vs. individual) might affect boredom and as a consequence the self-control that is needed to adhere to the activity. Finally, the feedback and reward structure of a sport or exercise (e.g., the availability and immediacy of a success like scoring a goal) is likely to influence whether it gives rise to the experience of boredom.

These propositions raise the question of how to measure boredom within the various exercise contexts. There are well-established self-report measures of domain-unspecific trait (Struk et al., 2017) and state boredom (Fahlman et al., 2013, for an overview, see Vodanovich and Watt, 2016). However, as boredom is a highly contextualized experience (Chin et al., 2017) it makes sense to assess it with reference to the specific context (Vodanovich and Watt, 2016). In the exercise domain, the recently developed *Bored of Sports Scale* (BOSS; Wolff et al., 2020a) already allows researchers to assess individual differences in exercise-related boredom (example item: “Exercising is dull and monotonous”) (Wolff et al., 2020a). Overall, there is a need to adapt and develop further sport specific questionnaires to measure boredom experienced in specific sport settings (e.g., boredom during long-distance runs), during specific exercise activities (e.g., jogging), and in the distinct settings of individual vs. collective activities (e.g., gym workouts vs. team sports).

Boredom has additional substantive implications for research in sports and exercise that only peripherally affect task-induced self-control demands. For instance, when running there may be several moments when the mind is engaged with unrelated thoughts—a phenomenon referred to as mind-wandering (Smallwood and Schooler, 2006; Christoff et al., 2018). Mind-wandering has become a highly researched topic in the past few decades (Callard et al., 2013) and has been shown to be related to boredom (Isacescu et al., 2017; Martarelli et al., 2020). Indeed, both experiences signal that a current task is not engaging one’s attentional resources fully. Mind-wandering might occur when the actual experience is boring. When one cannot change overt behavior, an alternative that is always available is to explore inner worlds (Mills and Christoff, 2018). Like boredom, mind-wandering has only scarcely been addressed in sports science (Latinjak, 2018). However, it is a relevant concept, because especially deliberate forms of mind-wandering might be used as a strategy to counteract boredom. On the other hand, spontaneous mind-wandering might derail attentional engagement with an exercise and thereby further exacerbate boredom and the challenge of continuing the activity. Moreover, as is the case with the dynamics of boredom and self-control, mind-wandering likely also changes over time and should not be considered as a static experience (Christoff et al., 2016). Investigating boredom and related constructs in a sports context is relevant not only for understanding their impact on sports engagement and performance but also as a potential avenue to improve participation rates in sports activities by helping individuals to better regulate their engagement with these healthful activities. In the same vein, as some people utilize sport and exercise to alleviate boredom (Morris et al., 2003), it is crucial to understand the social (e.g., exercising in a group as opposed to exercising alone) and contextual (e.g., participating in virtual bike racing, as opposed to simply pedaling alone on the hometrainer) conditions that make sports and exercise a powerful remedy for boredom.

To conclude, boredom is omnipresent in everyday life, and the sports and exercise context is no exception. We call for investigating *when* and *why* boredom occurs in self-control research in sports and exercise, and *how* it affects goal-directed behavior in these settings.

## Data Availability Statement

The datasets presented in this study can be found in online repositories. The names of the repository/repositories and accession number(s) can be found below: https://osf.io/6uc2k/.

## Ethics Statement

Ethical review and approval was not required for the study on human participants in accordance with the local legislation and institutional requirements. The patients/participants provided their written informed consent to participate in this study.

## Author Contributions

WW, MB, CM, and JD developed the ideas presented here. WW wrote the first draft of the manuscript. WW, MB, CD, and JD revised the manuscript. All authors contributed to the article and approved the submitted version.

## Conflict of Interest

The authors declare that the research was conducted in the absence of any commercial or financial relationships that could be construed as a potential conflict of interest.
